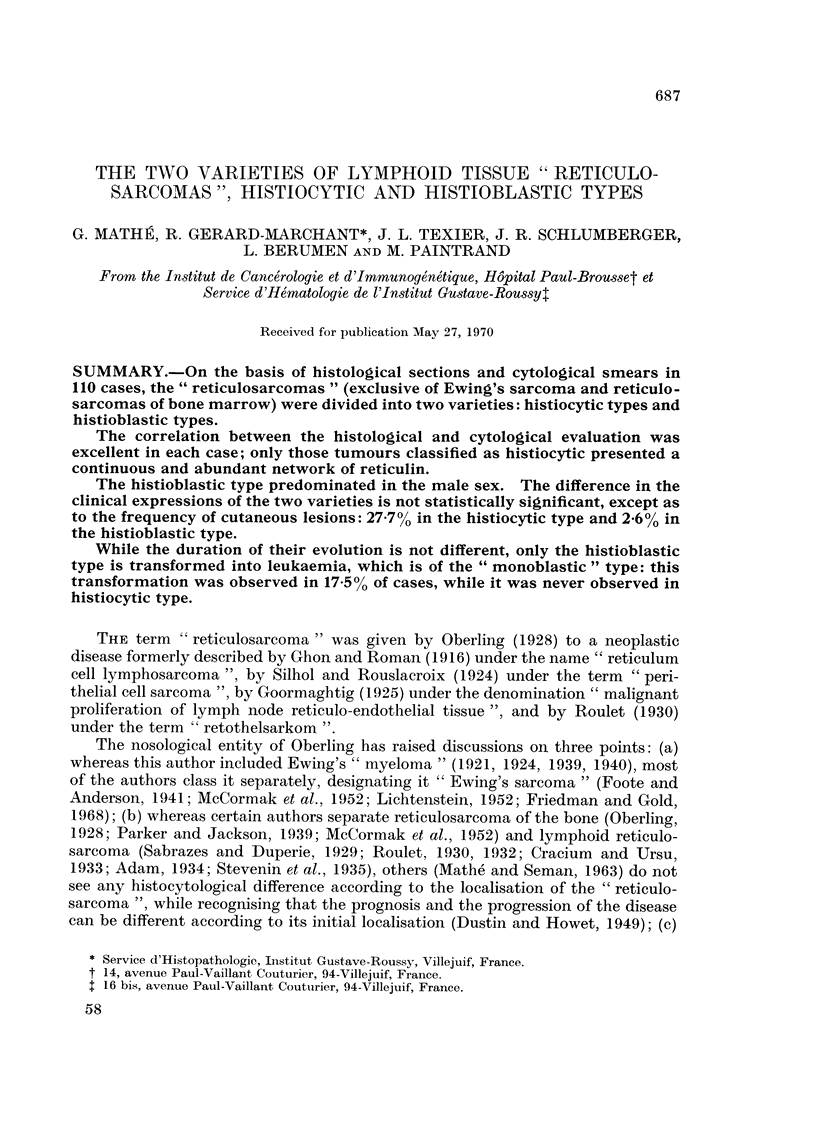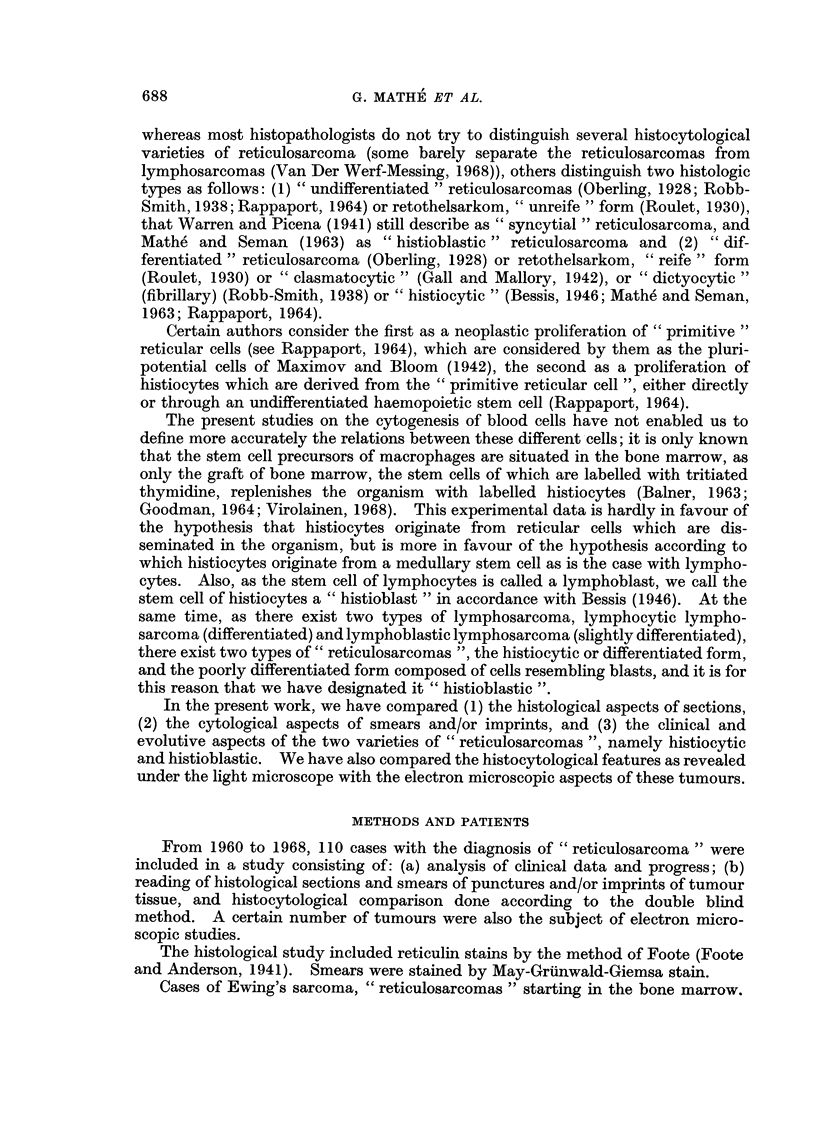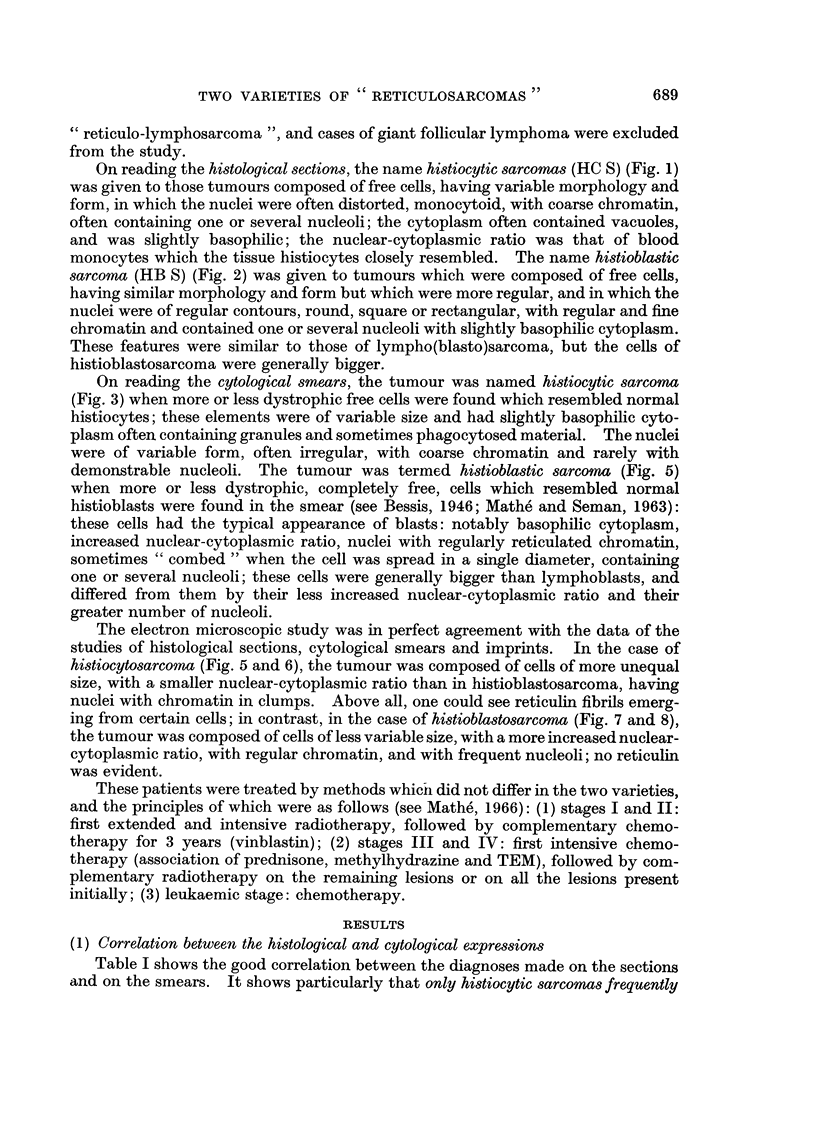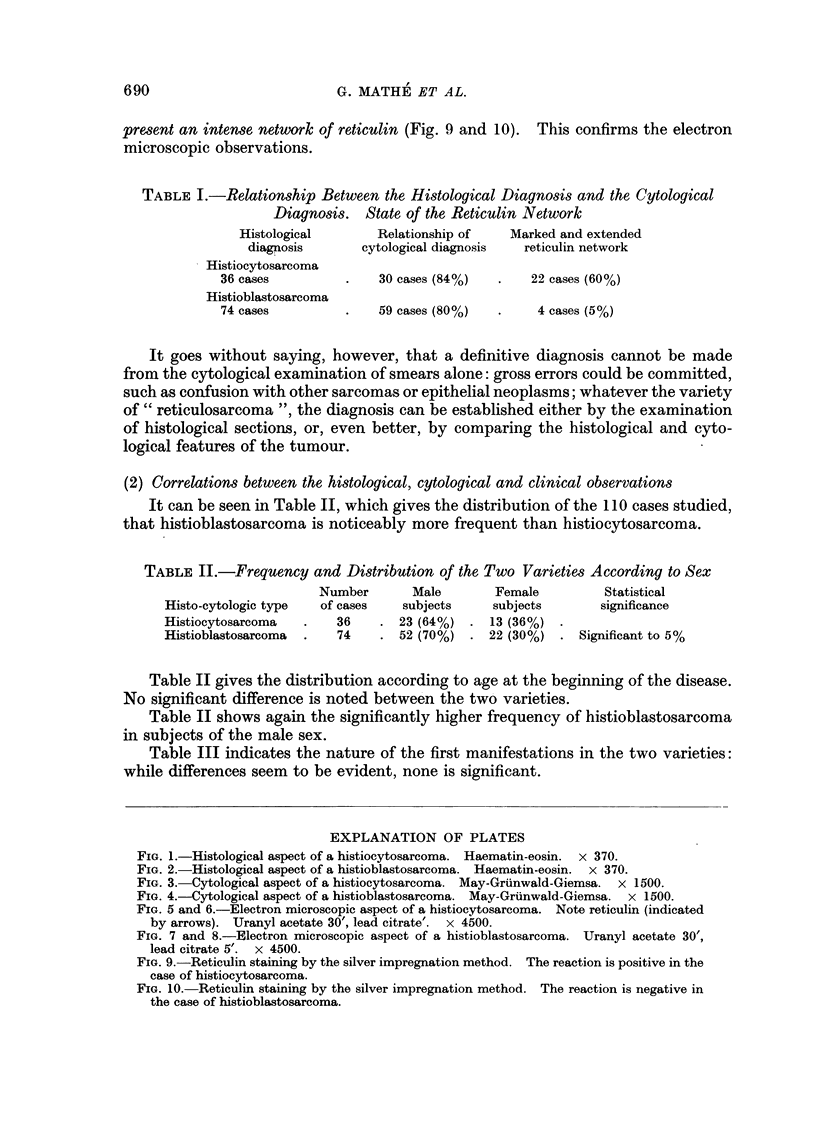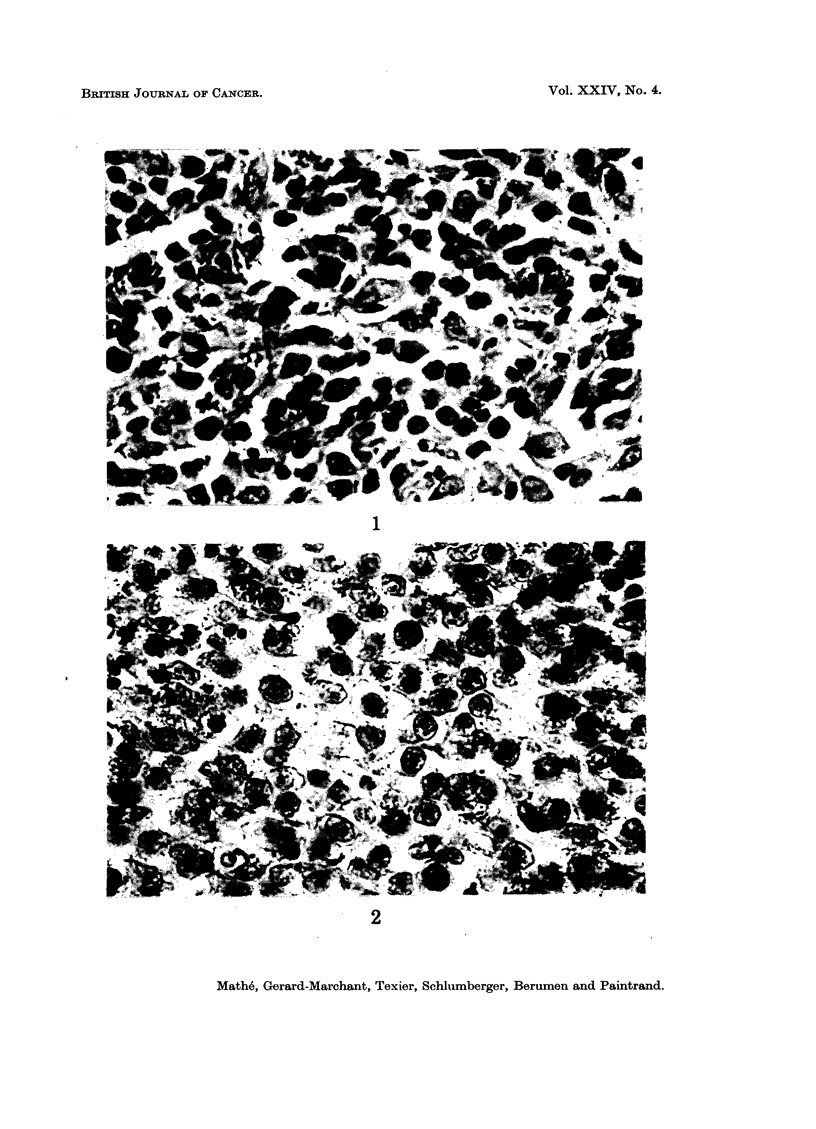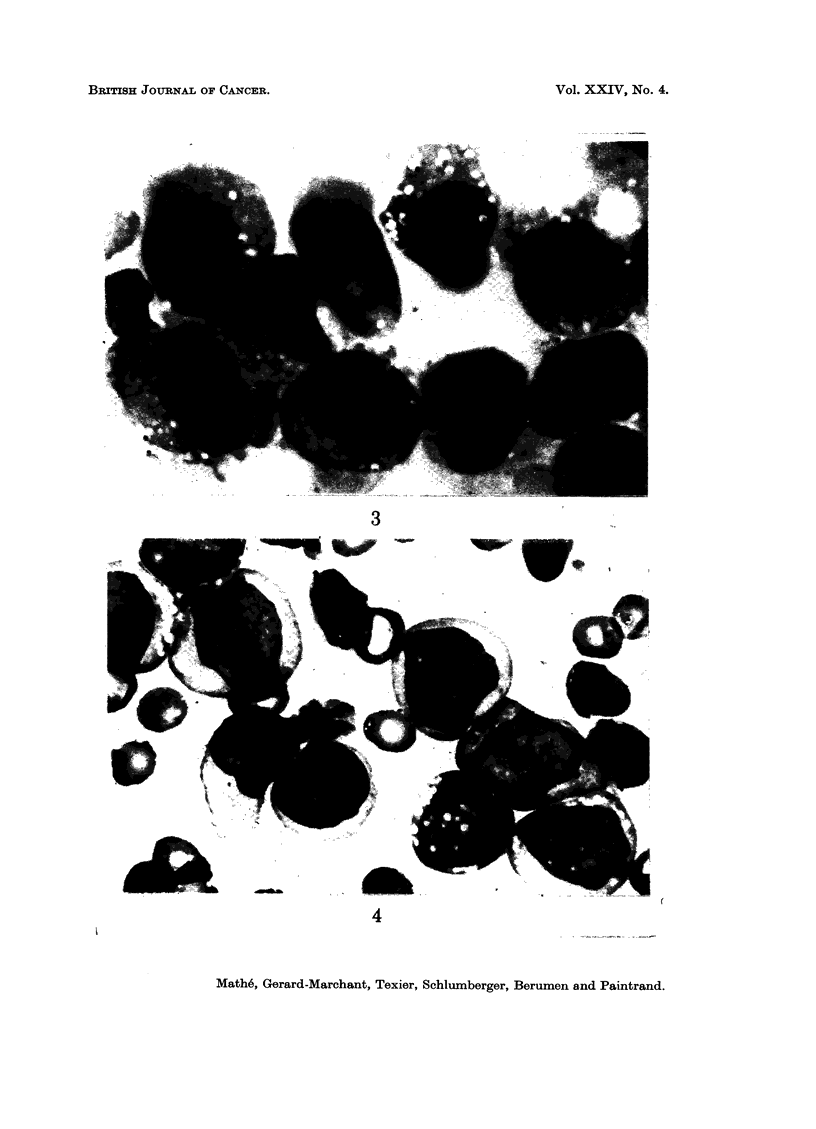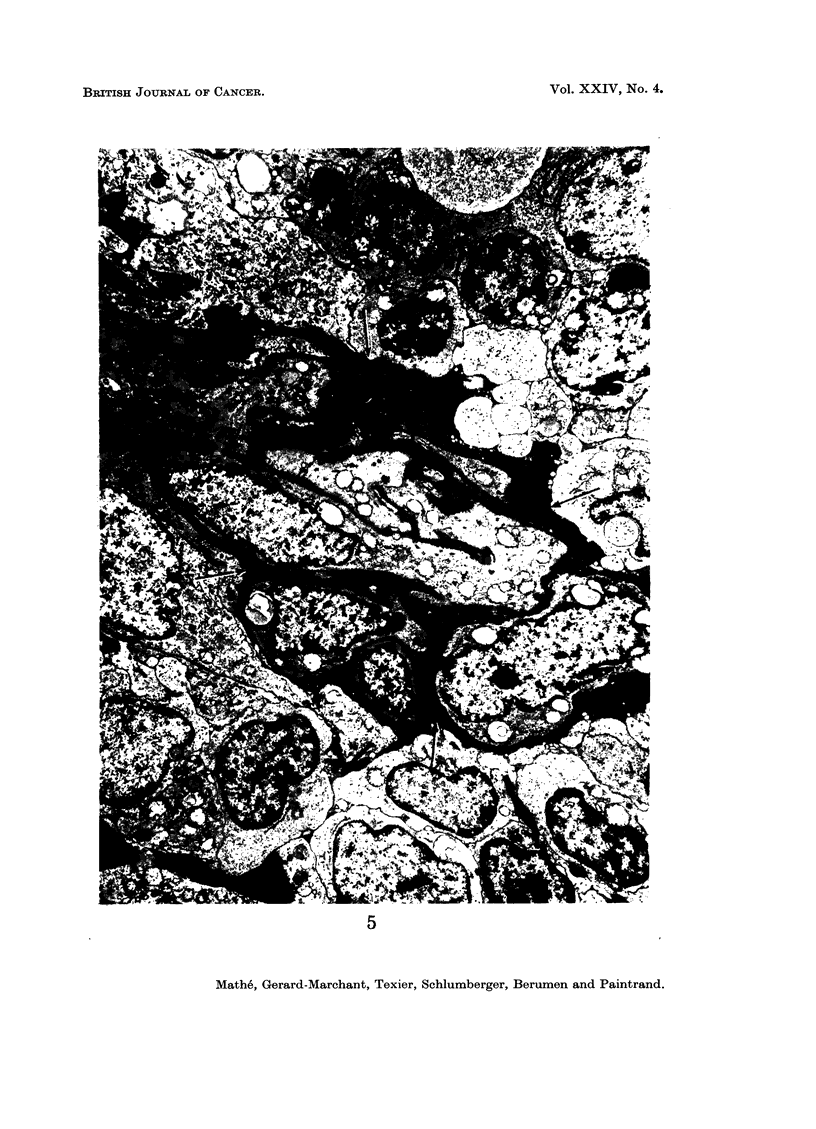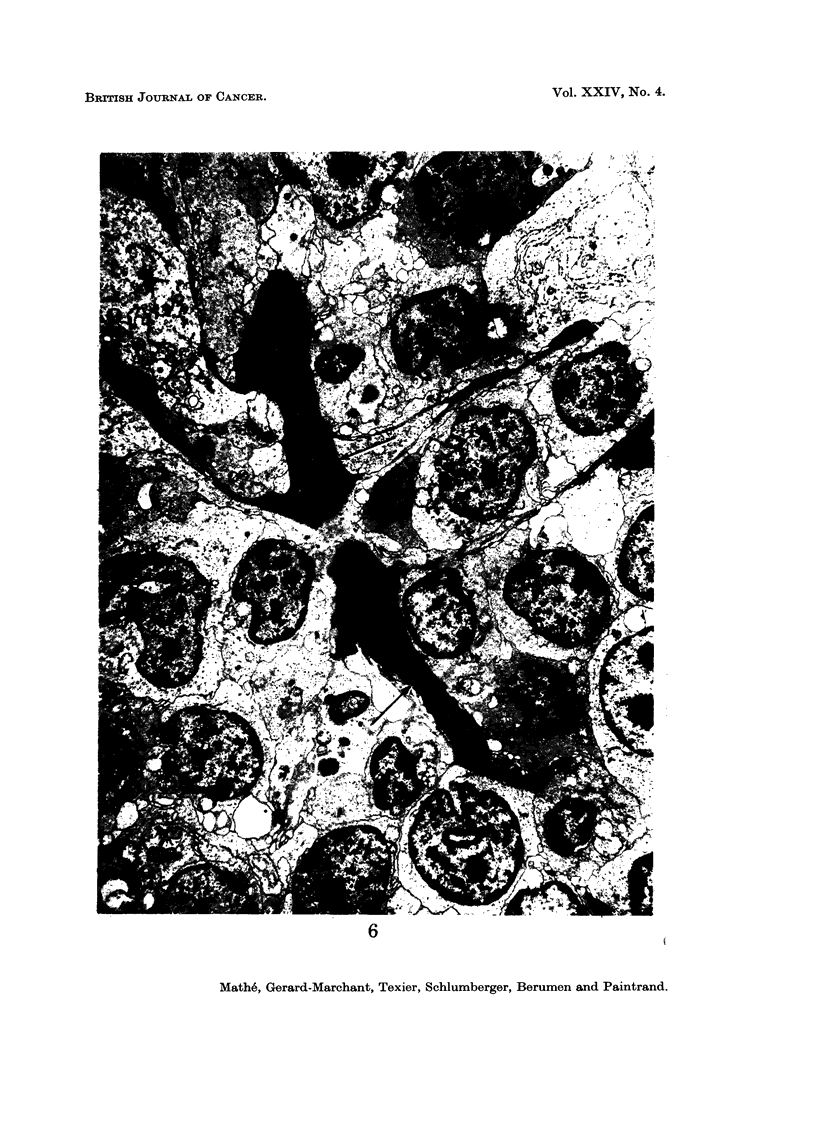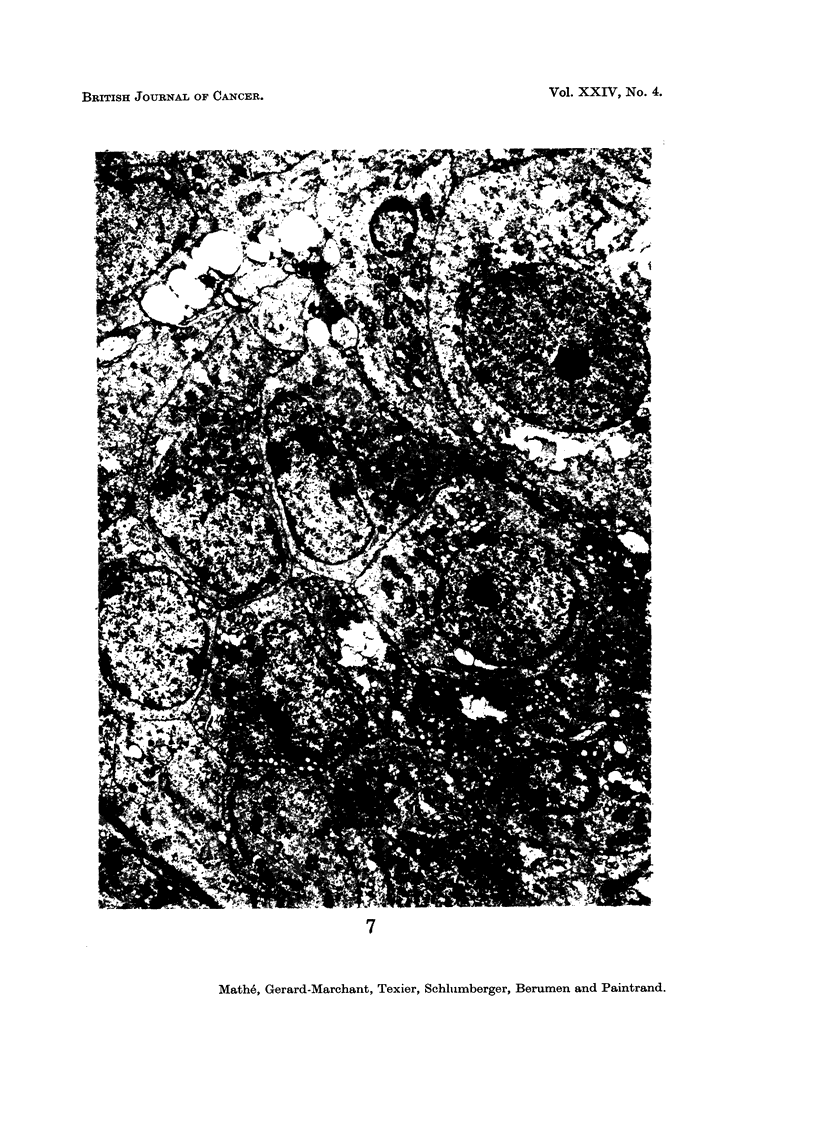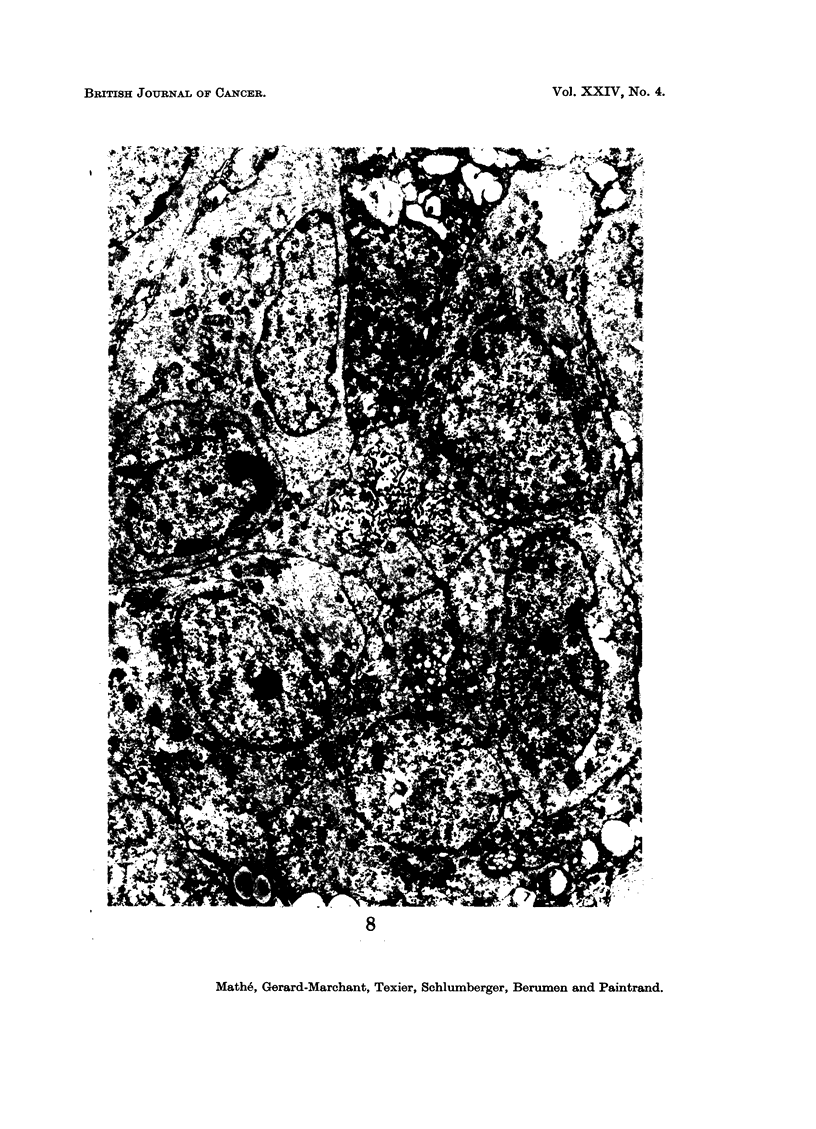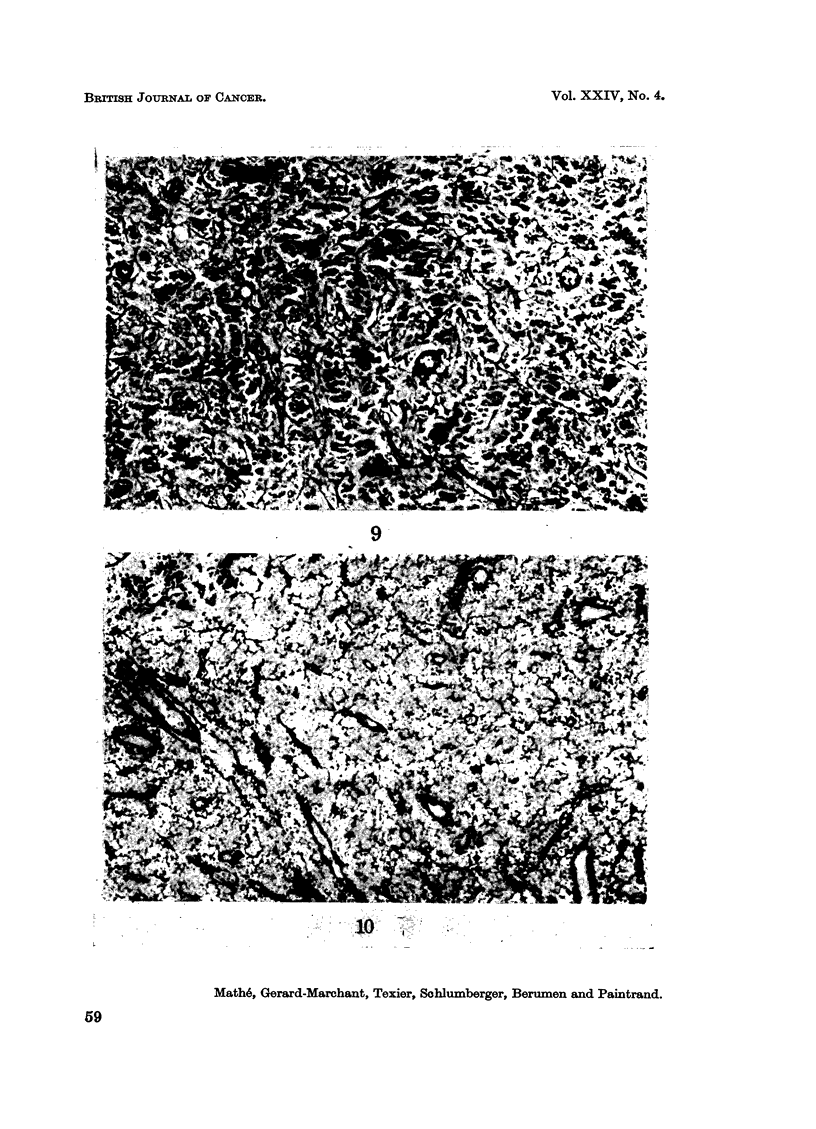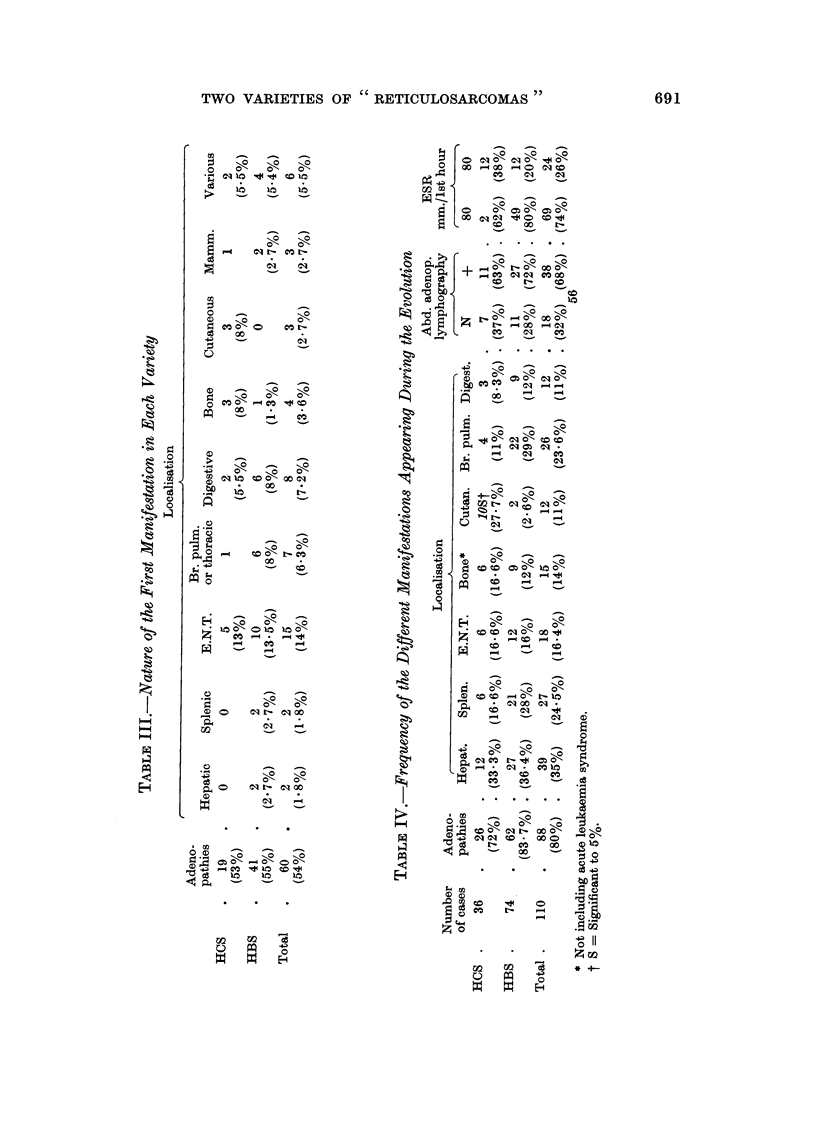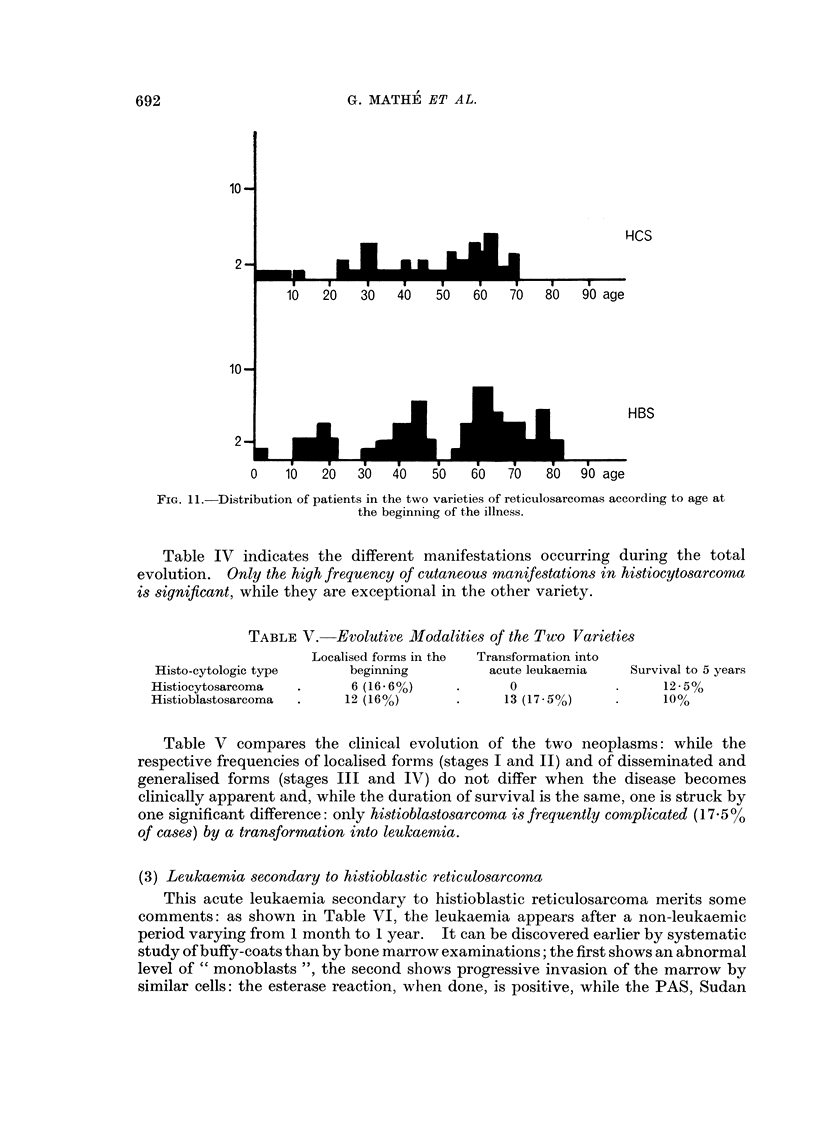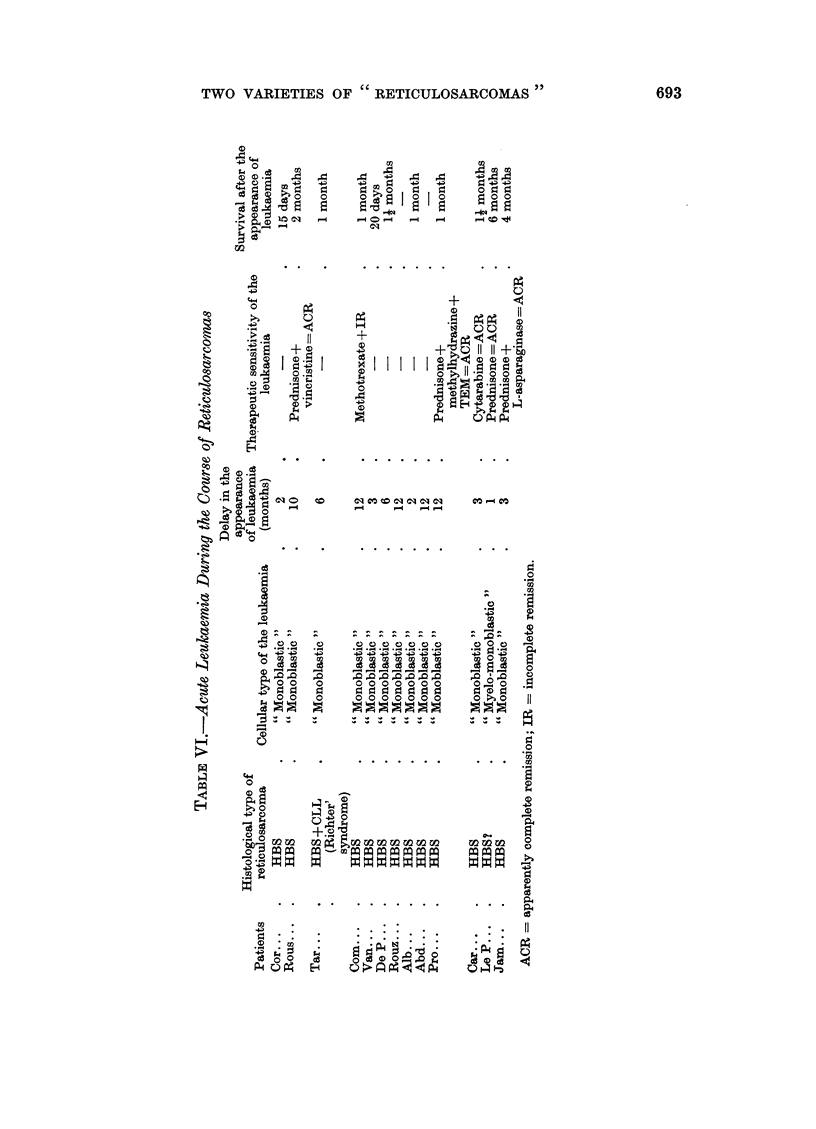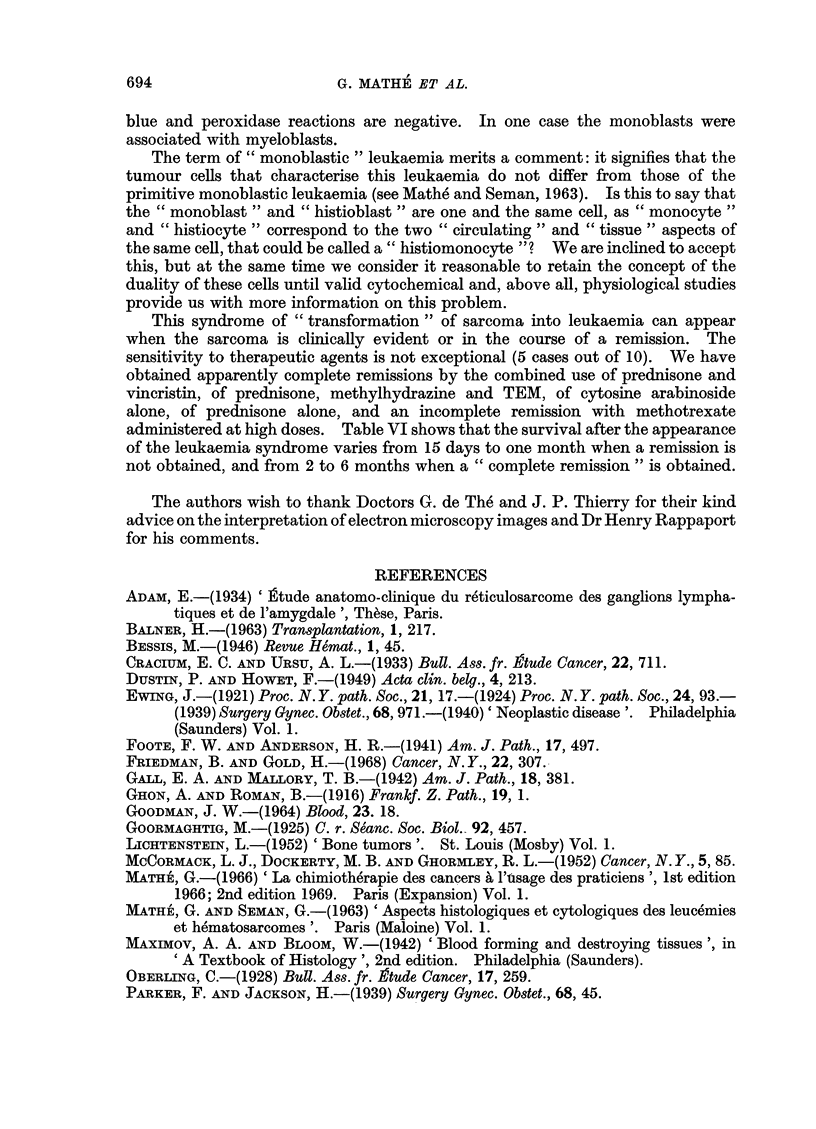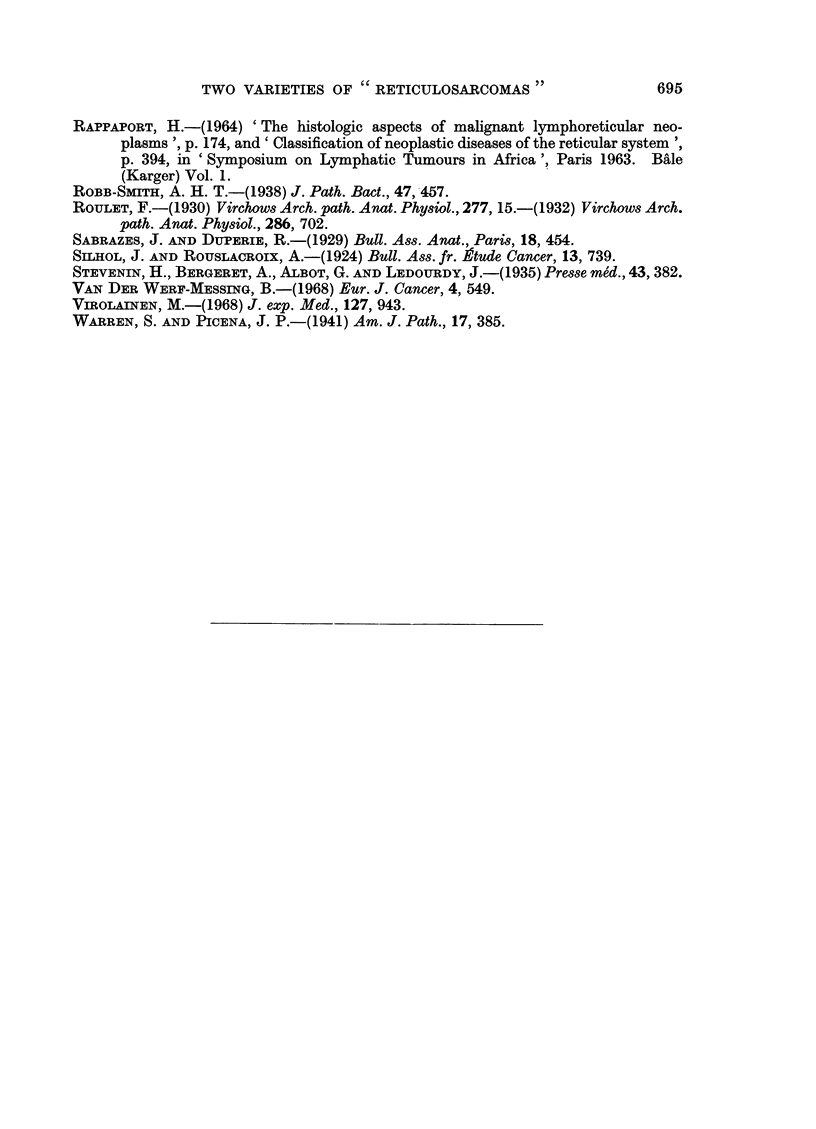# The Two Varieties of Lymphoid Tissue “Reticulosarcomas” Histiocytic and Histioblastic Types

**DOI:** 10.1038/bjc.1970.82

**Published:** 1970-12

**Authors:** G. Mathé, R. Gerard-Marchant, J. L. Texier, J. R. Schlumberger, L. Berumen, M. Paintrand

## Abstract

**Images:**


### THE TWO VARIETIES OF LYMPHOID TISSUE " RETICULO-SARCOMAS ", HISTIOCYTIC AND HISTIOBLASTIC TYPES

G. MATHÉ, R. GERARD-MARCHANT*, J. L. TEXIER, J. R. SCHLUMBERGER, L. BERUMEN AND M. PAINTRAND

*From the Institut de Cancérologie et d'Immunogénétique, Hôpital Paul-Brousse† et Service d'Hématologie de l'Institut Gustave-Roussy‡*

Received for publication May 27, 1970

**SUMMARY.—On the basis of histological sections and cytological smears in 110 cases, the " reticulosarcomas " (exclusive of Ewing's sarcoma and reticulosarcomas of bone marrow) were divided into two varieties: histiocytic types and histioblastic types.**

**The correlation between the histological and cytological evaluation was excellent in each case; only those tumours classified as histiocytic presented a continuous and abundant network of reticulin.**

**The histioblastic type predominated in the male sex. The difference in the clinical expressions of the two varieties is not statistically significant, except as to the frequency of cutaneous lesions: 27·7% in the histiocytic type and 2·6% in the histioblastic type.**

**While the duration of their evolution is not different, only the histioblastic type is transformed into leukaemia, which is of the " monoblastic " type: this transformation was observed in 17·5% of cases, while it was never observed in histiocytic type.**

THE term " reticulosarcoma " was given by Oberling (1928) to a neoplastic disease formerly described by Ghon and Roman (1916) under the name " reticulum cell lymphosarcoma ", by Silhol and Rouslacroix (1924) under the term " perithelial cell sarcoma ", by Goormaghtig (1925) under the denomination " malignant proliferation of lymph node reticulo-endothelial tissue ", and by Roulet (1930) under the term " retothelsarkom ".

The nosological entity of Oberling has raised discussions on three points: (a) whereas this author included Ewing's " myeloma " (1921, 1924, 1939, 1940), most of the authors class it separately, designating it " Ewing's sarcoma " (Foote and Anderson, 1941; McCormak *et al.*, 1952; Lichtenstein, 1952; Friedman and Gold, 1968); (b) whereas certain authors separate reticulosarcoma of the bone (Oberling, 1928; Parker and Jackson, 1939; McCormak *et al.*, 1952) and lymphoid reticulosarcoma (Sabrazes and Duperie, 1929; Roulet, 1930, 1932; Cracium and Ursu, 1933; Adam, 1934; Stevenin *et al.*, 1935), others (Mathé and Seman, 1963) do not see any histocytological difference according to the localisation of the " reticulosarcoma ", while recognising that the prognosis and the progression of the disease can be different according to its initial localisation (Dustin and Howet, 1949); (c)

* Service d'Histopathologie, Institut Gustave-Roussy, Villejuif, France.
† 14, avenue Paul-Vaillant Couturier, 94-Villejuif, France.
‡ 16 bis, avenue Paul-Vaillant Couturier, 94-Villejuif, France.

whereas most histopathologists do not try to distinguish several histocytological varieties of reticulosarcoma (some barely separate the reticulosarcomas from lymphosarcomas (Van Der Werf-Messing, 1968)), others distinguish two histologic types as follows: (1) " undifferentiated " reticulosarcomas (Oberling, 1928; Robb-Smith, 1938; Rappaport, 1964) or retothelsarkom, " unreife " form (Roulet, 1930), that Warren and Picena (1941) still describe as " syncytial " reticulosarcoma, and Mathé and Seman (1963) as " histioblastic " reticulosarcoma and (2) " differentiated " reticulosarcoma (Oberling, 1928) or retothelsarkom, " reife " form (Roulet, 1930) or " clasmatocytic " (Gall and Mallory, 1942), or " dictyocytic " (fibrillary) (Robb-Smith, 1938) or " histiocytic " (Bessis, 1946; Mathé and Seman, 1963; Rappaport, 1964).

Certain authors consider the first as a neoplastic proliferation of " primitive " reticular cells (see Rappaport, 1964), which are considered by them as the pluripotential cells of Maximov and Bloom (1942), the second as a proliferation of histiocytes which are derived from the " primitive reticular cell ", either directly or through an undifferentiated haemopoietic stem cell (Rappaport, 1964).

The present studies on the cytogenesis of blood cells have not enabled us to define more accurately the relations between these different cells; it is only known that the stem cell precursors of macrophages are situated in the bone marrow, as only the graft of bone marrow, the stem cells of which are labelled with tritiated thymidine, replenishes the organism with labelled histiocytes (Balner, 1963; Goodman, 1964; Virolainen, 1968). This experimental data is hardly in favour of the hypothesis that histiocytes originate from reticular cells which are disseminated in the organism, but is more in favour of the hypothesis according to which histiocytes originate from a medullary stem cell as is the case with lymphocytes. Also, as the stem cell of lymphocytes is called a lymphoblast, we call the stem cell of histiocytes a " histioblast " in accordance with Bessis (1946). At the same time, as there exist two types of lymphosarcoma, lymphocytic lymphosarcoma (differentiated) and lymphoblastic lymphosarcoma (slightly differentiated), there exist two types of " reticulosarcomas ", the histiocytic or differentiated form, and the poorly differentiated form composed of cells resembling blasts, and it is for this reason that we have designated it " histioblastic ".

In the present work, we have compared (1) the histological aspects of sections, (2) the cytological aspects of smears and/or imprints, and (3) the clinical and evolutive aspects of the two varieties of " reticulosarcomas ", namely histiocytic and histioblastic. We have also compared the histocytological features as revealed under the light microscope with the electron microscopic aspects of these tumours.

### METHODS AND PATIENTS

From 1960 to 1968, 110 cases with the diagnosis of " reticulosarcoma " were included in a study consisting of: (a) analysis of clinical data and progress; (b) reading of histological sections and smears of punctures and/or imprints of tumour tissue, and histocytological comparison done according to the double blind method. A certain number of tumours were also the subject of electron microscopic studies.

The histological study included reticulin stains by the method of Foote (Foote and Anderson, 1941). Smears were stained by May-Grünwald-Giemsa stain.

Cases of Ewing's sarcoma, " reticulosarcomas " starting in the bone marrow.

" reticulo-lymphosarcoma ", and cases of giant follicular lymphoma were excluded from the study.

On reading the *histological sections*, the name *histiocytic sarcomas* (HC S) (Fig. 1) was given to those tumours composed of free cells, having variable morphology and form, in which the nuclei were often distorted, monocytoid, with coarse chromatin, often containing one or several nucleoli; the cytoplasm often contained vacuoles, and was slightly basophilic; the nuclear-cytoplasmic ratio was that of blood monocytes which the tissue histiocytes closely resembled. The name *histioblastic sarcoma* (HB S) (Fig. 2) was given to tumours which were composed of free cells, having similar morphology and form but which were more regular, and in which the nuclei were of regular contours, round, square or rectangular, with regular and fine chromatin and contained one or several nucleoli with slightly basophilic cytoplasm. These features were similar to those of lympho(blasto)sarcoma, but the cells of histioblastosarcoma were generally bigger.

On reading the *cytological smears*, the tumour was named *histiocytic sarcoma* (Fig. 3) when more or less dystrophic free cells were found which resembled normal histiocytes; these elements were of variable size and had slightly basophilic cytoplasm often containing granules and sometimes phagocytosed material. The nuclei were of variable form, often irregular, with coarse chromatin and rarely with demonstrable nucleoli. The tumour was termed *histioblastic sarcoma* (Fig. 5) when more or less dystrophic, completely free, cells which resembled normal histioblasts were found in the smear (see Bessis, 1946; Mathé and Seman, 1963): these cells had the typical appearance of blasts: notably basophilic cytoplasm, increased nuclear-cytoplasmic ratio, nuclei with regularly reticulated chromatin, sometimes " combed " when the cell was spread in a single diameter, containing one or several nucleoli; these cells were generally bigger than lymphoblasts, and differed from them by their less increased nuclear-cytoplasmic ratio and their greater number of nucleoli.

The electron microscopic study was in perfect agreement with the data of the studies of histological sections, cytological smears and imprints. In the case of *histiocytosarcoma* (Fig. 5 and 6), the tumour was composed of cells of more unequal size, with a smaller nuclear-cytoplasmic ratio than in histioblastosarcoma, having nuclei with chromatin in clumps. Above all, one could see reticulin fibrils emerging from certain cells; in contrast, in the case of *histioblastosarcoma* (Fig. 7 and 8), the tumour was composed of cells of less variable size, with a more increased nuclear-cytoplasmic ratio, with regular chromatin, and with frequent nucleoli; no reticulin was evident.

These patients were treated by methods which did not differ in the two varieties, and the principles of which were as follows (see Mathé, 1966): (1) stages I and II: first extended and intensive radiotherapy, followed by complementary chemotherapy for 3 years (vinblastin); (2) stages III and IV: first intensive chemotherapy (association of prednisone, methylhydrazine and TEM), followed by complementary radiotherapy on the remaining lesions or on all the lesions present initially; (3) leukaemic stage: chemotherapy.

### RESULTS

### (1) *Correlation between the histological and cytological expressions*

Table I shows the good correlation between the diagnoses made on the sections and on the smears. It shows particularly that *only histiocytic sarcomas frequently*

*present an intense network of reticulin* (Fig. 9 and 10). This confirms the electron microscopic observations.

TABLE I.—*Relationship Between the Histological Diagnosis and the Cytological Diagnosis. State of the Reticulin Network*

| Histological diagnosis | Relationship of cytological diagnosis | Marked and extended reticulin network |
|---|---|---|
| Histiocytosarcoma 36 cases | 30 cases (84%) | 22 cases (60%) |
| Histioblastosarcoma 74 cases | 59 cases (80%) | 4 cases (5%) |

It goes without saying, however, that a definitive diagnosis cannot be made from the cytological examination of smears alone: gross errors could be committed, such as confusion with other sarcomas or epithelial neoplasms; whatever the variety of " reticulosarcoma ", the diagnosis can be established either by the examination of histological sections, or, even better, by comparing the histological and cytological features of the tumour.

(2) *Correlations between the histological, cytological and clinical observations*

It can be seen in Table II, which gives the distribution of the 110 cases studied, that histioblastosarcoma is noticeably more frequent than histiocytosarcoma.

TABLE II.—*Frequency and Distribution of the Two Varieties According to Sex*

| Histo-cytologic type | Number of cases | Male subjects | Female subjects | Statistical significance |
|---|---|---|---|---|
| Histiocytosarcoma | 36 | 23 (64%) | 13 (36%) | |
| Histioblastosarcoma | 74 | 52 (70%) | 22 (30%) | Significant to 5% |

Table II gives the distribution according to age at the beginning of the disease. No significant difference is noted between the two varieties.

Table II shows again the significantly higher frequency of histioblastosarcoma in subjects of the male sex.

Table III indicates the nature of the first manifestations in the two varieties: while differences seem to be evident, none is significant.

---

EXPLANATION OF PLATES

FIG. 1.—Histological aspect of a histiocytosarcoma. Haematin-eosin. × 370.
FIG. 2.—Histological aspect of a histioblastosarcoma. Haematin-eosin. × 370.
FIG. 3.—Cytological aspect of a histiocytosarcoma. May-Grünwald-Giemsa. × 1500.
FIG. 4.—Cytological aspect of a histioblastosarcoma. May-Grünwald-Giemsa. × 1500.
FIG. 5 and 6.—Electron microscopic aspect of a histiocytosarcoma. Note reticulin (indicated by arrows). Uranyl acetate 30′, lead citrate′. × 4500.
FIG. 7 and 8.—Electron microscopic aspect of a histioblastosarcoma. Uranyl acetate 30′, lead citrate 5′. × 4500.
FIG. 9.—Reticulin staining by the silver impregnation method. The reaction is positive in the case of histiocytosarcoma.
FIG. 10.—Reticulin staining by the silver impregnation method. The reaction is negative in the case of histioblastosarcoma.

1

2

Mathé, Gerard-Marchant, Texier, Schlumberger, Berumen and Paintrand.

3

4

Mathé, Gerard-Marchant, Texier, Schlumberger, Berumen and Paintrand.

5

Mathé, Gerard-Marchant, Texier, Schlumberger, Berumen and Paintrand.

6

Mathé, Gerard-Marchant, Texier, Schlumberger, Berumen and Paintrand.

7

Mathé, Gerard-Marchant, Texier, Schlumberger, Berumen and Paintrand.

8

Mathé, Gerard-Marchant, Texier, Schlumberger, Berumen and Paintrand.

9

10

Mathé, Gerard-Marchant, Texier, Schlumberger, Berumen and Paintrand.

TABLE III.—*Nature of the First Manifestation in Each Variety*

| | Adeno-pathies | Localisation: Hepatic | Splenic | E.N.T. | Br. pulm. or thoracic | Digestive | Bone | Cutaneous | Mamm. | Various |
|---|---|---|---|---|---|---|---|---|---|---|
| HCS . | 19 (53%) . | 0 | 0 | 5 (13%) | 1 | 2 (5·5%) | 3 (8%) | 3 (8%) | 1 | 2 (5·5%) |
| HBS . | 41 (55%) . | 2 (2·7%) | 2 (2·7%) | 10 (13·5%) | 6 (8%) | 6 (8%) | 1 (1·3%) | 0 | 2 (2·7%) | 4 (5·4%) |
| Total . | 60 (54%) . | 2 (1·8%) | 2 (1·8%) | 15 (14%) | 7 (6·3%) | 8 (7·2%) | 4 (3·6%) | 3 (2·7%) | 3 (2·7%) | 6 (5·5%) |

TABLE IV.—*Frequency of the Different Manifestations Appearing During the Evolution*

| | Number of cases | Adeno-pathies | Localisation: Hepat. | Splen. | E.N.T. | Bone* | Cutan. | Br. pulm. | Digest. | Abd. adenop. lymphography: N | + | ESR mm./1st hour: 80 | 80 |
|---|---|---|---|---|---|---|---|---|---|---|---|---|---|
| HCS . | 36 | 26 (72%) | 12 (33·3%) | 6 (16·6%) | 6 (16·6%) | 6 (16·6%) | *10*S† (27·7%) | 4 (11%) | 3 (8·3%) | 7 (37%) | 11 (63%) | 2 (62%) | 12 (38%) |
| HBS . | 74 | 62 (83·7%) | 27 (36·4%) | 21 (28%) | 12 (16%) | 9 (12%) | 2 (2·6%) | 22 (29%) | 9 (12%) | 11 (28%) | 27 (72%) | 49 (80%) | 12 (20%) |
| Total . | 110 | 88 (80%) | 39 (35%) | 27 (24·5%) | 18 (16·4%) | 15 (14%) | 12 (11%) | 26 (23·6%) | 12 (11%) | 18 (32%) | 38 (68%) | 69 (74%) | 24 (26%) |

* Not including acute leukaemia syndrome.

† S = Significant to 5%.

FIG. 11.—Distribution of patients in the two varieties of reticulosarcomas according to age at the beginning of the illness.

Table IV indicates the different manifestations occurring during the total evolution. *Only the high frequency of cutaneous manifestations in histiocytosarcoma is significant*, while they are exceptional in the other variety.

TABLE V.—*Evolutive Modalities of the Two Varieties*

| Histo-cytologic type | Localised forms in the beginning | Transformation into acute leukaemia | Survival to 5 years |
|---|---|---|---|
| Histiocytosarcoma | 6 (16·6%) | 0 | 12·5% |
| Histioblastosarcoma | 12 (16%) | 13 (17·5%) | 10% |

Table V compares the clinical evolution of the two neoplasms: while the respective frequencies of localised forms (stages I and II) and of disseminated and generalised forms (stages III and IV) do not differ when the disease becomes clinically apparent and, while the duration of survival is the same, one is struck by one significant difference: only *histioblastosarcoma is frequently complicated* (17·5% *of cases*) *by a transformation into leukaemia.*

(3) *Leukaemia secondary to histioblastic reticulosarcoma*

This acute leukaemia secondary to histioblastic reticulosarcoma merits some comments: as shown in Table VI, the leukaemia appears after a non-leukaemic period varying from 1 month to 1 year. It can be discovered earlier by systematic study of buffy-coats than by bone marrow examinations; the first shows an abnormal level of " monoblasts ", the second shows progressive invasion of the marrow by similar cells: the esterase reaction, when done, is positive, while the PAS, Sudan

TABLE VI.—*Acute Leukaemia During the Course of Reticulosarcomas*

| Patients | Histological type of reticulosarcoma | Cellular type of the leukaemia | Delay in the appearance of leukaemia (months) | Therapeutic sensitivity of the leukaemia | Survival after the appearance of leukaemia |
|---|---|---|---|---|---|
| Cor... | HBS | " Monoblastic " | 2 | — | 15 days |
| Rous... | HBS | " Monoblastic " | 10 | Prednisone + vincristine = ACR | 2 months |
| Tar... | HBS + CLL (Richter' syndrome) | " Monoblastic " | 6 | — | 1 month |
| Com... | HBS | " Monoblastic " | 12 | Methotrexate + IR | 1 month |
| Van... | HBS | " Monoblastic " | 3 | — | 20 days |
| De P... | HBS | " Monoblastic " | 6 | — | 1½ months |
| Rouz... | HBS | " Monoblastic " | 12 | — | — |
| Alb... | HBS | " Monoblastic " | 2 | — | 1 month |
| Abd... | HBS | " Monoblastic " | 12 | — | — |
| Pro... | HBS | " Monoblastic " | 12 | Prednisone + methylhydrazine + TEM = ACR | 1 month |
| Car... | HBS | " Monoblastic " | 3 | Cytarabine = ACR | 1½ months |
| Le P... | HBS? | " Myelo-monoblastic " | 1 | Prednisone = ACR | 6 months |
| Jam... | HBS | " Monoblastic " | 3 | Prednisone + L-asparaginase = ACR | 4 months |

ACR = apparently complete remission; IR = incomplete remission.

blue and peroxidase reactions are negative. In one case the monoblasts were associated with myeloblasts.

The term of " monoblastic " leukaemia merits a comment: it signifies that the tumour cells that characterise this leukaemia do not differ from those of the primitive monoblastic leukaemia (see Mathé and Seman, 1963). Is this to say that the " monoblast " and " histioblast " are one and the same cell, as " monocyte " and " histiocyte " correspond to the two " circulating " and " tissue " aspects of the same cell, that could be called a " histiomonocyte "? We are inclined to accept this, but at the same time we consider it reasonable to retain the concept of the duality of these cells until valid cytochemical and, above all, physiological studies provide us with more information on this problem.

This syndrome of " transformation " of sarcoma into leukaemia can appear when the sarcoma is clinically evident or in the course of a remission. The sensitivity to therapeutic agents is not exceptional (5 cases out of 10). We have obtained apparently complete remissions by the combined use of prednisone and vincristin, of prednisone, methylhydrazine and TEM, of cytosine arabinoside alone, of prednisone alone, and an incomplete remission with methotrexate administered at high doses. Table VI shows that the survival after the appearance of the leukaemia syndrome varies from 15 days to one month when a remission is not obtained, and from 2 to 6 months when a " complete remission " is obtained.

The authors wish to thank Doctors G. de Thé and J. P. Thierry for their kind advice on the interpretation of electron microscopy images and Dr Henry Rappaport for his comments.

### REFERENCES

ADAM, E.—(1934) ' Étude anatomo-clinique du réticulosarcome des ganglions lymphatiques et de l'amygdale ', Thèse, Paris.

BALNER, H.—(1963) *Transplantation*, **1**, 217.

BESSIS, M.—(1946) *Revue Hémat.*, **1**, 45.

CRACIUM, E. C. AND URSU, A. L.—(1933) *Bull. Ass. fr. Étude Cancer*, **22**, 711.

DUSTIN, P. AND HOWET, F.—(1949) *Acta clin. belg.*, **4**, 213.

EWING, J.—(1921) *Proc. N.Y. path. Soc.*, **21**, 17.—(1924) *Proc. N.Y. path. Soc.*, **24**, 93.—(1939) *Surgery Gynec. Obstet.*, **68**, 971.—(1940) ' Neoplastic disease '. Philadelphia (Saunders) Vol. 1.

FOOTE, F. W. AND ANDERSON, H. R.—(1941) *Am. J. Path.*, **17**, 497.

FRIEDMAN, B. AND GOLD, H.—(1968) *Cancer, N.Y.*, **22**, 307.

GALL, E. A. AND MALLORY, T. B.—(1942) *Am. J. Path.*, **18**, 381.

GHON, A. AND ROMAN, B.—(1916) *Frankf. Z. Path.*, **19**, 1.

GOODMAN, J. W.—(1964) *Blood*, **23**. 18.

GOORMAGHTIG, M.—(1925) *C. r. Séanc. Soc. Biol.*. **92**, 457.

LICHTENSTEIN, L.—(1952) ' Bone tumors '. St. Louis (Mosby) Vol. 1.

MCCORMACK, L. J., DOCKERTY, M. B. AND GHORMLEY, R. L.—(1952) *Cancer, N.Y.*, **5**, 85.

MATHÉ, G.—(1966) ' La chimiothérapie des cancers à l'usage des praticiens ', 1st edition 1966; 2nd edition 1969. Paris (Expansion) Vol. 1.

MATHÉ, G. AND SEMAN, G.—(1963) ' Aspects histologiques et cytologiques des leucémies et hématosarcomes '. Paris (Maloine) Vol. 1.

MAXIMOV, A. A. AND BLOOM, W.—(1942) ' Blood forming and destroying tissues ', in ' A Textbook of Histology ', 2nd edition. Philadelphia (Saunders).

OBERLING, C.—(1928) *Bull. Ass. fr. Étude Cancer*, **17**, 259.

PARKER, F. AND JACKSON, H.—(1939) *Surgery Gynec. Obstet.*, **68**, 45.

RAPPAPORT, H.—(1964) 'The histologic aspects of malignant lymphoreticular neoplasms', p. 174, and 'Classification of neoplastic diseases of the reticular system', p. 394, in 'Symposium on Lymphatic Tumours in Africa', Paris 1963. Bâle (Karger) Vol. 1.
ROBB-SMITH, A. H. T.—(1938) *J. Path. Bact.*, **47**, 457.
ROULET, F.—(1930) *Virchows Arch. path. Anat. Physiol.*, **277**, 15.—(1932) *Virchows Arch. path. Anat. Physiol.*, **286**, 702.
SABRAZES, J. AND DUPERIE, R.—(1929) *Bull. Ass. Anat., Paris*, **18**, 454.
SILHOL, J. AND ROUSLACROIX, A.—(1924) *Bull. Ass. fr. Étude Cancer*, **13**, 739.
STEVENIN, H., BERGERET, A., ALBOT, G. AND LEDOURDY, J.—(1935) *Presse méd.*, **43**, 382.
VAN DER WERF-MESSING, B.—(1968) *Eur. J. Cancer*, **4**, 549.
VIROLAINEN, M.—(1968) *J. exp. Med.*, **127**, 943.
WARREN, S. AND PICENA, J. P.—(1941) *Am. J. Path.*, **17**, 385.